# Selection and Validation of Stable Reference Genes for RT-qPCR in *Scotogramma trifolii* (Lepidoptera: Noctuidae)

**DOI:** 10.3390/insects16050527

**Published:** 2025-05-15

**Authors:** Anpei Yang, Hang Zhang, Weiwei Bai, Ruifeng Ding, Weipeng Li, Guangkuo Li

**Affiliations:** 1 Institute of Plant Protection, Xinjiang Uygur Autonomous Region Academy of Agricultural Sciences, Urumqi 830091, China; yap2002@126.com (A.Y.); 4zhanghang@163.com (H.Z.); hebaige@163.com (W.B.); drf022@163.com (R.D.); 13279007407@163.com (W.L.); 2 Key Laboratory of Integrated Pest Management on Crops in Northwestern Oasis, Ministry of Agriculture and Rural Affairs, National Plant Protection Scientific Observation and Experiment Station of Korla, Urumqi 830091, China

**Keywords:** *Scotogramma trifolii*, reference genes, gene expression, normalization, RT-qPCR

## Abstract

The clover cutworm, *Scotogramma trifolii* Rottemberg (Lepidoptera: Noctuidae), is a globally distributed polyphagous pest causing significant economic losses to agricultural crops. RT-qPCR is a gold-standard technique for gene expression analysis, yet its accuracy depends critically on stable reference genes for data normalization. To address the lack of validated reference genes in *S. trifolii*, we evaluated six candidate genes (*β-actin*, *RPL9*, *GAPDH*, *RPL10*, *EF1-α*, and *TUB*) across four developmental stages (egg, larva, pupa, and adult) and six adult tissues (head, thorax, abdomen, wings, legs, and antennae) using geNorm, NormFinder, BestKeeper, and RefFinder algorithms. Stability analysis identified *β-actin*, *RPL9*, and *GAPDH* as the most reliable reference genes for developmental stage normalization, while *RPL10*, *GAPDH*, and *TUB* were validated for adult tissues. Functional validation using the odorant receptor gene *StriOR20* revealed significant discrepancies in relative expression levels when normalized with unstable reference genes (*TUB* and *RPL9*), emphasizing the necessity of rigorous reference gene selection. This study establishes the first comprehensive reference gene panel for *S. trifolii*, providing a robust foundation for gene expression studies in this agriculturally important pest.

## Simple Summary

Real-time quantitative polymerase chain reaction (RT-qPCR) is a powerful technique for quantifying gene expression, with the selection of stable reference genes being critical for accurate normalization of expression data. In this study, we systematically evaluated the expression stability of six candidate reference genes (*β-actin*, *RPL9*, *GAPDH*, *RPL10*, *EF1-α*, and *TUB*) across four developmental stages (egg, larva, pupa, and adult) and six adult tissues (head, thorax, abdomen, wings, legs, and antennae) of *Scotogramma trifolii* using four algorithmic tools (geNorm, NormFinder, BestKeeper, and RefFinder). Our results identified *β-actin*, *RPL9*, and *GAPDH* as the most stable reference genes for developmental stage normalization, while *RPL10*, *GAPDH*, and *TUB* were validated for adult tissues. Functional validation using the odorant receptor gene *StriOR20* demonstrated significant differences in relative expression levels when normalized with unstable reference genes (*TUB* and *RPL9*), underscoring the importance of appropriate reference gene selection. This study establishes the first validated set of reference genes for *S. trifolii*, providing a foundational resource for future gene expression studies in this agriculturally important pest species.

## 1. Introduction

*Scotogramma trifolii* Rottemberg, a globally distributed polyphagous leaf-feeding pest [1], exhibits highly mobile larvae with metastatic feeding behavior [2]. It is a partially explosive pest in northern China and has a wide range of hosts, feeding on more than 20 types of crops in 8 families and 27 types of weeds and causing damage to *Beta vulgaris*, *Gossypium herbaceum*, *Linum usitatissimum*, *Solanum tuberosum*, *Arachis hypogaea*, *Zea mays*, *Helianthus annuus*, *Ricinus communis*, *Glycine max*, *Triticum aestivum*, and cruciferous vegetables, thus threatening food security and the sustainable development of agriculture [3,4]. RT-qPCR is a cornerstone technique in molecular biology for quantifying gene expression across diverse biological contexts [5,6,7]. Renowned for its high sensitivity, specificity, and reproducibility [8], RT-qPCR remains susceptible to multiple confounding variables that can compromise data accuracy. Key sources of variability include RNA quality and quantity, primer specificity and amplification efficiency, cDNA synthesis efficiency, PCR reaction conditions, and experimental variability [9,10,11,12,13,14,15]. To address these challenges, normalization using stably expressed reference genes serves as a critical strategy to mitigate experimental noise and ensure reliable target gene quantification [16,17,18,19].

In lepidopteran gene expression studies, commonly employed reference genes include glyceraldehyde-3-phosphate dehydrogenase (*GAPDH*), *β-actin*, translation elongation factor 1α (*TEF-1α*), phospholipase A2 (*PLA2*), arginine kinase (*AK*), tubulin (*TUB*), TATA-binding protein (*TBP*), and ribosomal proteins (*RPLs*) [20,21,22,23,24,25,26]. While these genes are often assumed to exhibit constitutive expression across experimental conditions [27,28], the current “reference genes” are not stable under different experimental conditions and samples, and homologous reference genes show inconsistent expression stability under the same experimental conditions in different species [29]. Therefore, it is necessary to evaluate the stability of reference genes under different conditions.

In this study, we selected six candidate genes of *S. trifoli*, including *GAPDH*, *RPL9, RPL10*, *EF1-α*, *TUB*, and *β-actin*, and evaluated their expression stability in a spatiotemporal manner using four software programs, including geNorm v3.5, NormFinder v0.953, BestKeeper, and RefFinder. Furthermore, we assessed the expression profiles of odorant receptor genes (*StriOR20*) to verify the accuracy and reliability of the selected reference genes. This study will provide stable reference genes for analyzing the gene expression in *S. trifolii*.

## 2. Materials and Methods

### 2.1. Insect Rearing

The *S. trifolii* specimens were originally collected from sugar beet fields in Changji City, Xinjiang Uygur Autonomous Region, China, and have been successfully maintained for over 20 generations under controlled laboratory conditions at the Institute of Plant Protection, Xinjiang Academy of Agricultural Sciences (Urumqi, China). Larval stages were reared on a standardized artificial diet, while adult populations were maintained in mesh-rearing cages with ad libitum access to 10% honey water. All life stages were cultured under controlled environmental parameters: 26 ± 1 °C temperature, 70 ± 5% relative humidity, and a 16L:8D photoperiod.

### 2.2. Experimental Design and Sample Collection

Biological specimens were categorized into the following two experimental cohorts: developmental stages (egg, larva, pupa, and adult) and adult tissue types (head, thorax, abdomen, wing, leg, and antenna). Five biological replicates were established for each sample category. Post-collection, specimens were immediately transferred to 1.5 mL microcentrifuge tubes, subjected to liquid nitrogen flash-freezing, and archived at −80 °C until use in downstream assays.

#### 2.2.1. Developmental State

Biological specimens were classified into four developmental modules: eggs, larval instars (1st–6th), sex-specific pupae (male and female), and sex-specific adults (male and female). For each biological replicate, sample sizes were standardized as follows: 30 eggs, 20 first-instar larvae, 10 second-instar larvae, and one individual each for third- to sixth-instar larvae, male pupae, female pupae, unmated male adults, and unmated female adults. All samples were randomly selected from a single population, yielding a total of 55 specimens (11 developmental modules × 5 replicates).

#### 2.2.2. Adult Tissues

Adult tissue samples were dissected as follows: antennae (15 pairs per replicate), antennae-removed heads (2 individuals), wing-/leg-free thorax (1 individual), whole abdomens (1 individual), wings (3 individuals), and legs (3 individuals). Each of the six tissue types was dissected under stereomicroscopy, immediately transferred to 1.5 mL microcentrifuge tubes, and stored on dry ice during collection. This yielded 30 specimens in total (6 tissues × 5 biological replicates).

### 2.3. Total RNA Extraction and cDNA First Strand Synthesis

Total RNA isolation from each sample was performed using the *TransZol* Up Plus RNA Kit (ER501-01-V2, TransGen Biotech, Beijing, China) following the manufacturer’s protocol. Post-isolation, RNA concentration and purity were quantified using a NanoDrop 2000c spectrophotometer (Thermo Fisher Scientific, Waltham, MA, USA). First-strand cDNA synthesis was conducted using 1 μg of total RNA with the *EasyScript*^®^ One-Step gDNA Removal and cDNA Synthesis SuperMix (AE311, TransGen Biotech, Beijing, China) according to the supplier’s guidelines. Following cDNA synthesis, reactions were immediately stored at −20 °C until use in RT-qPCR analyses.

### 2.4. Candidate Reference Genes and Primer Design

Six candidate reference genes (*GAPDH*, *RPL9*, *RPL10*, *EF1-α*, *TUB*, and *β-actin*) were selected from *S. trifolii* transcriptomic datasets (unpublished) based on common usage in insect gene expression studies. Primers for amplifying internal reference gene fragments were designed using Primer Premier 5.0 (Premier Biosoft International, Palo Alto, CA, USA) (Table 1), while locus-specific RT-qPCR primer pairs were developed using Beacon Designer 8.0 (Bio-Rad, Hercules, CA, USA) from validated transcript sequences (Table 2). All primers were commercially synthesized by Sangon Biotech (Shanghai, China), and the resulting RT-PCR amplicon sequences were deposited in GenBank under accession numbers PV394231-PV394236.

### 2.5. Standard Curve Construction and RT-qPCR

To determine primer efficiency and specificity, a 6-point 5-fold serial dilution series (1/5, 1/25, 1/125, 1/625, 1/3125, and 1/15625) of standard cDNA template was employed to generate RT-qPCR standard curves for each primer pair. Amplification efficiency (E) was calculated using the formula *E* = (10^(−1/slope)^ − 1) × 100 [30]. Expression profiling of reference genes was conducted using the QuantStudio 6 Flex Real-Time PCR System (Thermo Fisher Scientific). Reaction components included 10 μL of 2 × *PerfectStart*™ Green qPCR SuperMix (AQ601-01-V2, TransGen Biotech, Beijing, China), 0.4 μL each of forward and reverse primers (10 μM), and 1 μL of 5-fold diluted cDNA template. Thermal cycling conditions were 95 °C for 5 min, followed by 40 cycles of 95 °C for 15 s and 60 °C for 30 s. Three technical replicates were performed per biological replicate, with melt curve analysis included to verify amplicon specificity.

### 2.6. Stability Analysis

Expression stability of candidate reference genes was evaluated using four computational algorithms: geNorm v3.5 [13], NormFinder v0.953 [31], BestKeeper [32], and RefFinder [33] (https://www.ciidirsinaloa.com.mx/RefFinder-master/, accessed 21 February 2025). Stability metrics were calculated as follows: geNorm: M-values (lower values indicate higher stability); NormFinder: Stability values (SV; lower SV indicates better stability); BestKeeper: Standard deviation (SD; lower SD reflects higher stability); and RefFinder: Geometric mean (GM) of normalized ranks derived from the four algorithms. Optimal reference gene number was determined by geNorm pairwise variation analysis (Vn/Vn + 1). A threshold of 0.15 was applied: if Vn/Vn + 1 ≤ 0.15, n reference genes were deemed sufficient; otherwise, n + 1 genes were recommended for RT-qPCR normalization.

### 2.7. Stability Validation of Candidate Reference Genes

To validate the stability of top-ranked and least stable reference genes, odorant receptor 20 (*StriOR20*; GenBank: PV394237) was selected as the target gene. Relative expression levels of *StriOR20* were quantified using the 2^−ΔΔCt^ method [34] based on threshold cycle (Ct) values, with five biological replicates included in the analysis.

### 2.8. Statistical Analysis

Statistical analysis of reference gene stability values was performed using four computational tools. Significant differences were determined via one-way ANOVA followed by Tukey’s post hoc test in IBM SPSS Statistics 25.0 (IBM Corp., Armonk, NY, USA), with statistical significance set at *p* < 0.05. All data visualizations, including boxplots and heatmaps, were generated using GraphPad Prism 10.1.2 (GraphPad Software, San Diego, CA, USA).

## 3. Results

### 3.1. Specificity and Amplification Efficiency of RT-qPCR Primers

The primers for RT-qPCR were designed for six reference genes with amplification lengths of 79 bp (*GAPDH*) to 161 bp (*RPL9*) (Table 2). The results showed that the melting curves of the six reference genes were all single peaks (Figure S1), indicating the high specificity of the RT-qPCR primers. The amplification efficiencies of the primers ranged from 90.951% (*TUB*) to 108.777% (*RPL9*), and the regression coefficients (R^2^) ranged from 0.990 (*β-actin*, *GAPDH*, and *RPL9*) to 0.999 (*TUB*) (Table 2), indicating that the designed primers met the requirements of RT-qPCR experiments.

### 3.2. Expression Level of Six Candidate Reference Genes

**Figure 1:**
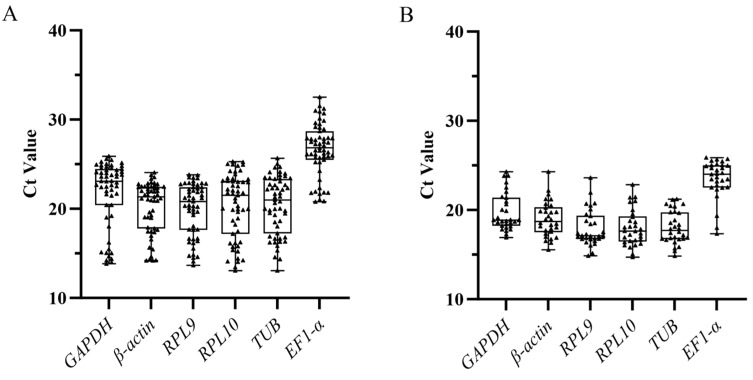
Expression levels of six candidate reference genes in the *S. trifolii*. (**A**) Development stage (n = 55). (**B**) Adult tissue (n = 30). The black-centered triangles represent the Ct values for each sample. The line across the box shows the median value. The top and bottom whiskers represent the 25th and 75th percentiles, respectively, and the bars represent the minimum and maximum.

The expression levels of the six candidate internal reference genes were evaluated using the Ct values obtained from RT-qPCR analysis. At different developmental stages, the expression of *RPL9* was the highest (Ct = 19.94); the expression of *EF1-α* was the lowest (Ct = 26.64); the expression of *β-actin* was the least variable (14.186–24.050) with a variation of 9.864; and the expression of *GAPDH* was the most variable (13.849–25.897) with a variation of 12.048. In adult tissues, the expression level of *RPL9* (Ct = 17.94) was the highest, while that of *EF1-α* (Ct = 23.50) was the lowest. *TUB* showed the lowest variation (14.837–21.213) with a variation of 6.376, and *β-actin* showed the highest variation (15.527–24.284) with a variation of 8.757 (Figure 1).

### 3.3. Expression Stability of Candidate Reference Genes

**Figure 2:**
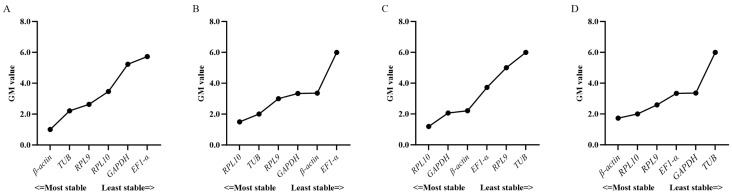
Comprehensive ranking of the expression stability of the six reference genes in the (**A**) egg, (**B**) larval, (**C**) pupa, and (**D**) adult stages as calculated by the RefFinder software.

**Figure 3:**
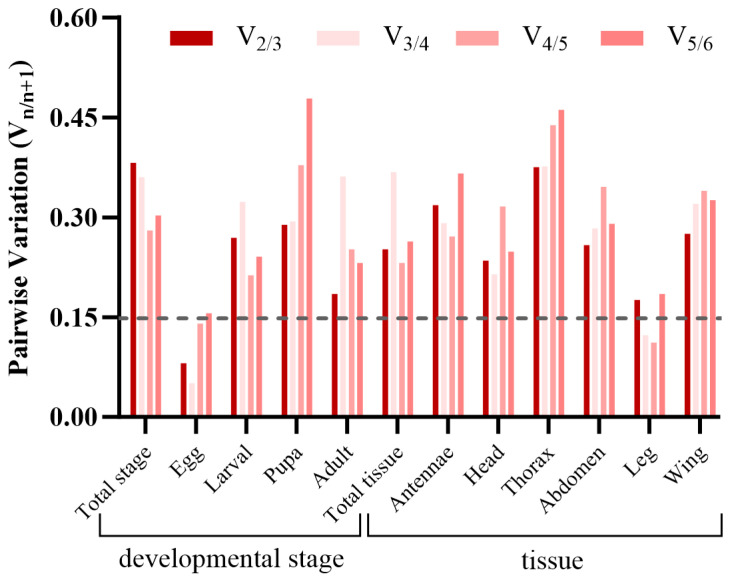
Optimal reference gene selection for RT-qPCR normalization in *S. trifolii* at different developmental stages and different tissue conditions. The threshold for pairwise variation (Vn/n + 1) is 0.15.

**Figure 4:**
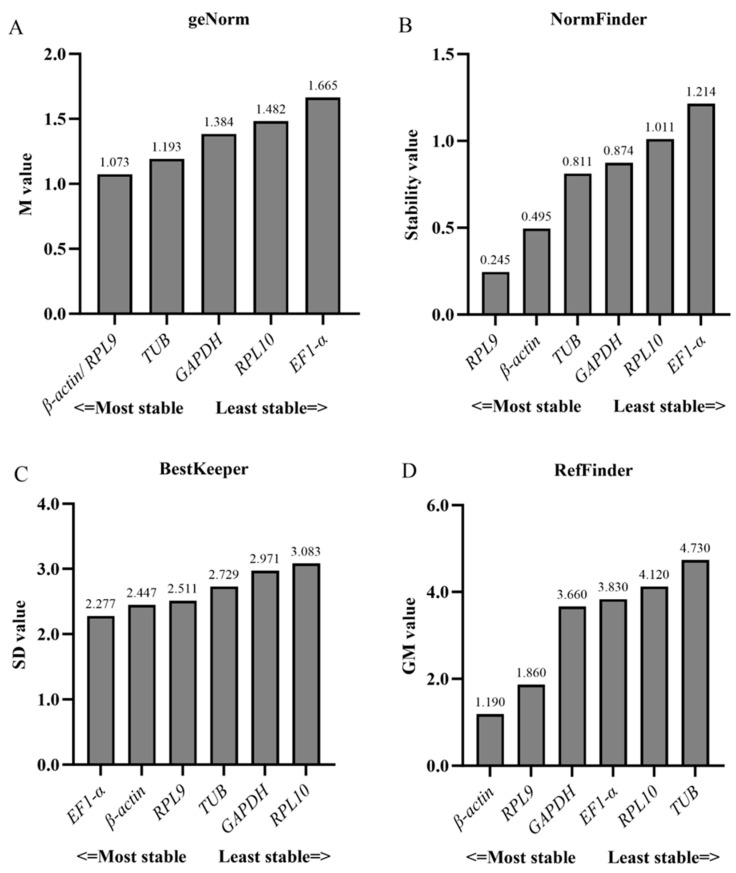
Expression stability of six candidate reference genes in *S. trifolii* across different developmental stages was evaluated using the following four computational tools: (**A**) geNorm, (**B**) NormFinder, (**C**) BestKeeper, and (**D**) RefFinder.

Based on RefFinder (Figure 2) and geNorm (Figure 3) analyses, the most stable reference genes in the egg stage were *β-actin* and *TUB*; *RPL10*, *TUB*, and *RPL9* were the most stable in the larval stage; *RPL10*, *GAPDH*, and *β-actin* were the most stable in the pupal stage; and *β-actin*, *RPL10*, and *RPL9* were the most stable in the adult stage. In all developmental stages, combined geNorm and NormFinder algorithms identified *β-actin* and *RPL9* as the two most stable reference genes. BestKeeper analysis independently validated *β-actin* and *EF1-α* as the most reliable normalizers. A comprehensive stability ranking generated by RefFinder demonstrated the following descending order: *β-actin* > *RPL9* > *GAPDH* > *EF1-α* > *RPL10* > *TUB* (Figure 4). geNorm analysis indicated a pairwise variation value of V2/3 = 0.381 (greater than the 0.15 threshold), supporting the use of three reference genes for accurate normalization. Based on this criterion and stability rankings, the optimal combination for developmental stage normalization was determined to be *β-actin*, *RPL9*, and *GAPDH* (Figure 3).

**Figure 5:**
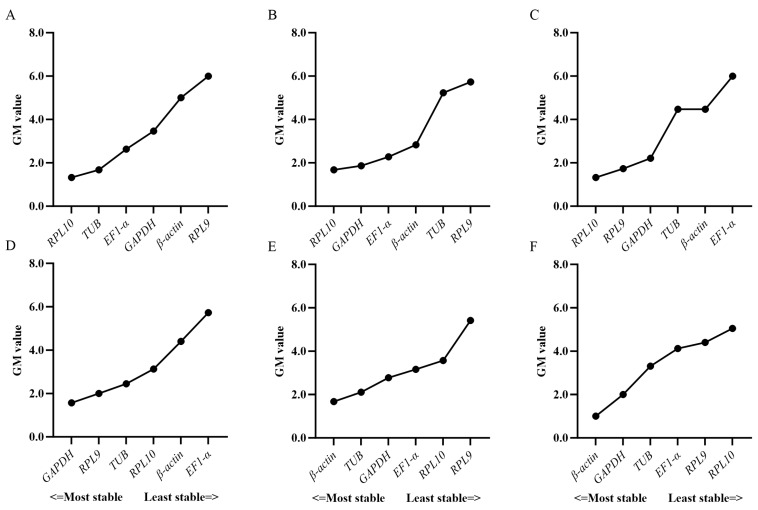
Comprehensive ranking of the expression stability of the six reference genes in six tissues of adult calculated according to the RefFinder software. (**A**) Antennae, (**B**) head, (**C**) thorax, (**D**) abdomen, (**E**) leg, and (**F**) wing.

**Figure 6:**
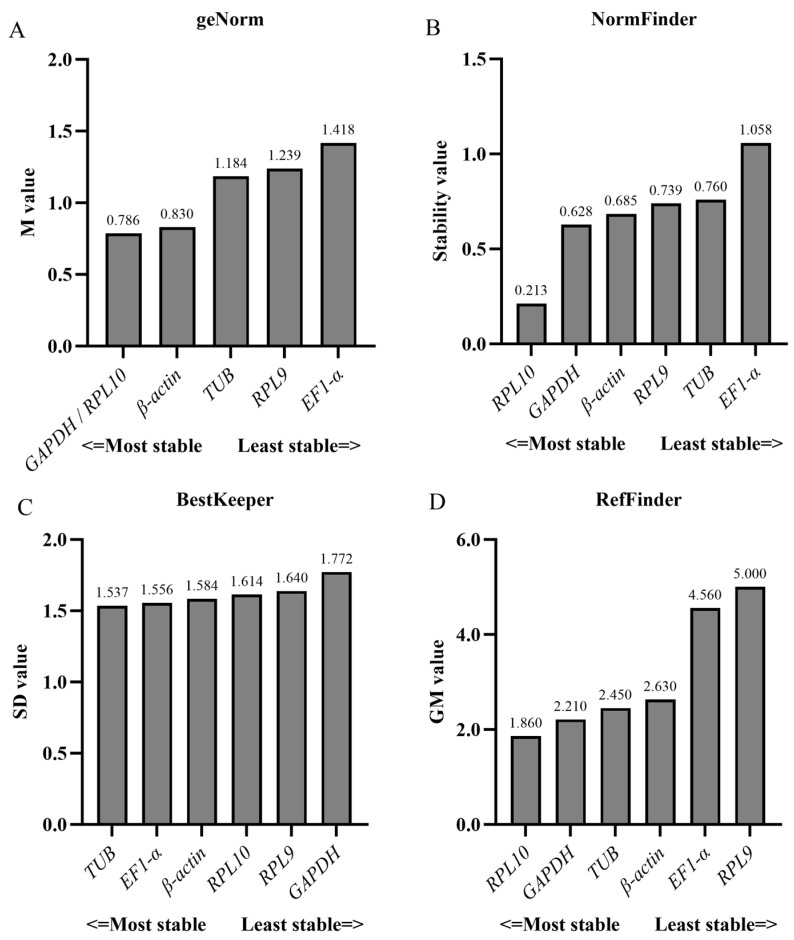
Expression stability of six candidate reference genes in *S. trifolii* adult tissues was evaluated using the following four distinct computational tools: (**A**) geNorm, (**B**) NormFinder, (**C**) BestKeeper, and (**D**) RefFinder.

According to the RefFinder and geNorm analyses of the expression stability of the reference genes in six adult tissues. *RPL10*, *TUB*, and *EF1-α* were the most stably expressed in the antennae; *RPL10*, *GAPDH*, and *EF1-α* were the most stably expressed in the head; *RPL10*, *RPL9*, and *GAPDH* were the most stably expressed in the thorax; *GAPDH*, *RPL9*, and *TUB* were the most stably expressed in the abdomen; *β-actin*, *TUB*, and *GAPDH* were the most stably expressed in the leg; and *β-actin*, *GAPDH*, and *TUB* were the most stably expressed in the wing (Figure 3 and Figure 5). In total adult tissues, geNorm identified *GAPDH* and *RPL9* as the most stable reference genes, NormFinder ranked *RPL10* highest, and BestKeeper validated *TUB* as the optimal normalizer. Finally, a comprehensive stability hierarchy derived from RefFinder demonstrated the following descending order: *RPL10* > *GAPDH* > TUB > *β-actin* > *EF1-α* > *RPL9* (Figure 6). geNorm analysis indicated a pairwise variation value of V2/3 = 0.252, which exceeds the 0.15 threshold for stability, supporting the inclusion of three reference genes. Specifically, *RPL10*, *GAPDH*, and *TUB* were selected for adult tissue normalization based on this criterion (Figure 3).

### 3.4. Verification of Candidate Reference Genes

**Figure 7:**
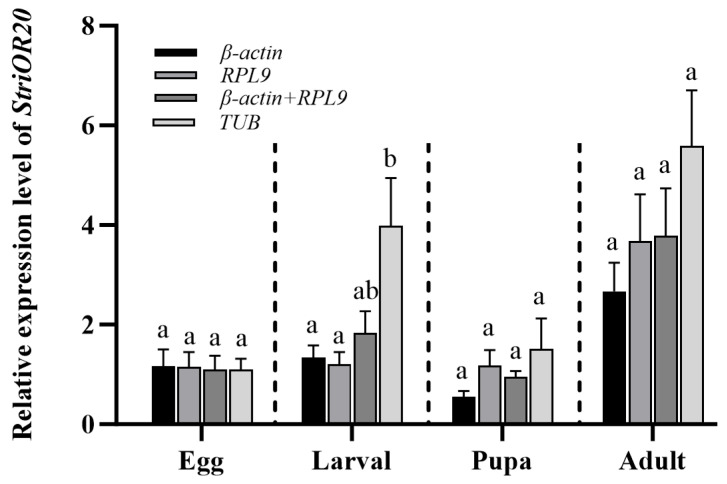
Expression level of *StriOR20* at different developmental stages of *S. trifolii*. Error bar, means ± standard error (SE) (n = 5). Different lowercase letters above bars in the same treatment represent significant differences (*p* < 0.05, one-way ANOVA analysis followed by Tukey’s test).

**Figure 8:**
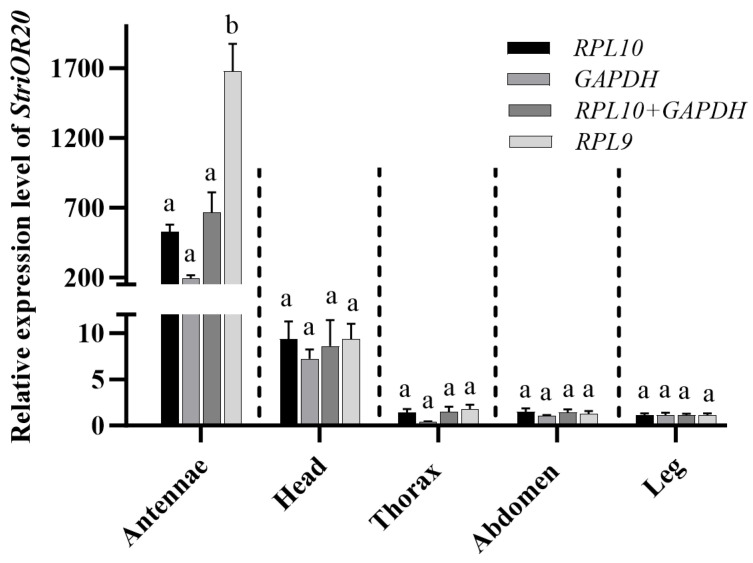
Expression level of *StriOR20* in different tissues of *Scotogramma trifolii*. Error bar, means ± SE (n = 5). Different lowercase letters above bars in the same treatment represent significant differences (*p* < 0.05, one-way ANOVA analysis followed by Tukey’s test).

To evaluate the effect of reference gene stability on gene expression quantification, the odorant receptor gene *StriOR20* from *S. trifolii* was selected as a target. Relative expression levels were quantified using the ΔΔCt method [34], comparing normalization results obtained with validated stable versus unstable reference genes across all the developmental stages and total adult tissues. There was a significant difference (*p* < 0.05) between the expression levels of *StriOR20* using the stable (*β-actin* and *RPL9*) and least stable reference genes (*TUB*) during the larval stage (Figure 7). No significant differences in *StriOR20* relative expression were detected between stable and unstable reference gene normalizations across egg, pupa, and adult stages. However, adult-stage expression normalized with unstable reference genes showed a 1.48–2.09-fold increase compared to stable normalization (Figure 7). In different tissues, *StriOR20* was highly expressed in the antennae, with low expression in other tissues. Antennae tissue showed a substantial difference (*p* < 0.001) in the relative expression levels of *StriOR20* when normalizing with stable reference genes (*RPL10* and *GAPDH*) compared to unstable ones (*RPL9*). In contrast, other tissues did not display any significant differences (Figure 8).

## 4. Discussion

RT-qPCR is the most widely used method to analyze gene expression; however, the accuracy and reliability of analyzing gene expression rely on stable reference genes for normalization. Current studies have shown that no reference gene is universally stably expressed, and the same reference gene in the same species appears to have different expression levels under different experimental conditions [29]; e.g., in this study, *RPL9* is the reference gene with stable expression at different developmental stages, but it is the most unstable under different tissue conditions. Meanwhile, the same internal reference gene also appeared to have different expression levels in different species under the same experimental conditions [29]. For example, *β-actin* was a stable reference gene at different developmental stages in *Plutella xylostella* [35], *Spodoptera exigua* [36], and *Mythimna separata* [37], and was the most unstable gene at different developmental stages in *Helicoverpa armigera* [38], *Spodoptera litura* [39], and *Spodoptera frugiperda* [29], which are less stable reference genes at different developmental stages. In order to obtain accurate experimental data for target genes, it is necessary to screen for reference genes that are stable under specific experimental conditions in specific species. Therefore, our study reported stable reference genes at different developmental stages and in different tissues of *S. trifolii*, which provides a basis for the expression study of target genes.

Commonly used software programs for stability analysis of reference genes are BestKeeper, geNorm, and NormFinder. However, the results of different software analyses are not consistent. Here, we found that at developmental stages, *EF1-α* was ranked lowest (least stable) in geNorm and NormFinder analyses, while BestKeeper was ranked highest (most stable). The differences between the results of BestKeeper and geNorm and NormFinder may be due to the different algorithms of the different programs, and similar findings have been reported in other insects [29,40,41]. Therefore, in order to eliminate the potential noise between the three algorithms, we used the online software RefFinder to rank the sorting of the three programs together as a final filter. In order to improve the reliability and accuracy of RT-qPCR data, researchers proposed to use multiple internal reference genes at the same time [13,35,42,43], and the exact number of suitable internal reference genes to be used was decided based on the pairwise variance value (Vn/n + 1) calculated by the geNorm software, which has a threshold value of 0.15. Vn/n + 1 was greater than 0.15 in all the results of this study, and we suggest that the number of suitable reference genes used is three.

In this study, *β-actin*, *RPL9*, and *GAPDH* were screened as stable reference genes at different developmental stages, and *RPL10*, *GAPDH*, and *TUB* were stable reference genes in different tissues. The results are similar to those reported in other insects, where *β-actin* is a stable reference gene at different developmental stages in *Leucinodes orbonalis* [44], *P. xylostella* [35], *S. exigua* [36], and *M. separata* [37]. *GAPDH* was the best reference gene for developmental stages in *Tuta absoluta* [45]. Meanwhile, *GAPDH* is the most stable reference gene in *S. litura* [39], *Sesamia inferens* [46], and *S. exigua* [36] under different tissue conditions, such as *TUB* and *RPL10* are stable in *S. frugiperda* [29], and *RPL10* is most stable in *S. litura* [39] and *T. absoluta* [45]. However, there are inconsistent reports in some species, such as *GAPDH* not being a stable reference gene in the developmental stages and tissues of *H. armigera* [43] and *S. frugiperda* [29], and *β-actin* being unstable in the developmental stages of *S. frugiperda* [29]. Meanwhile, in the present study, we found that the most stable reference genes at four developmental stages were not consistent and also differed from all developmental stages; the stability of expression of reference genes varied in six adult tissues and in total adult tissues, and similar studies have been reported in *S. exigua* [36]. Therefore, in future studies, we should select the appropriate reference genes according to the specific experimental requirements. The odorant receptor is an important chemosensory protein involved in the process of olfactory perception in insects and plays a very important role in the regulation of insect olfactory behavior [47,48]. In the present study, we verified the expression levels of *StriOR20* at different developmental stages and under different tissue treatments using stable and unstable reference genes. The results showed that the relative expression levels of *StriOR20* in the larval stage and antennae were significantly different when using stable and unstable reference genes. The same was reported in the validation of odorant-binding protein (*OBP*) expression in *S. frugiperda*, where significant differences in the expression patterns of *SfruOBP1* were observed when stable and unstable internal reference genes were used [29]. The present results further validate the importance of selecting appropriate stable reference genes in quantitative experiments for analyzing gene expression. Therefore, it is necessary to select and validate stable reference genes to improve the accuracy and reliability in the gene expression analysis of *S. trifolii*.

## 5. Conclusions

In this study, we analyzed the expression stability of six candidate reference genes in different developmental stages and different tissues of *S. trifolii* using RT-qPCR. We found that *β-actin*, *RPL9*, and *GAPDH* were reference genes stably expressed at different developmental stages, and *RPL10*, *GAPDH*, and *TUB* were reference genes with stable expression in different tissues. To the best of our knowledge, the results of this study are reported for the first time and provide a basis for gene expression analysis in *S. trifolii*.

## Figures and Tables

**Table 1:** Primers of reference genes in *S. trifolii*.

Gene	Primer Sequences (5′–3′)	Tm (°C)	Length (bp)
*TUB*	F:CCGACCTCTTCGTAGTCCTTCTC	60	1202
	R:ATGCCCTCGGACAAGACCC		
*β-actin*	F:TGTGTGACGAGGATGTTGCT	60	892
	R:GTGTTGGCGTACAGGTCCTT		
*RPL9*	F:CAAATAGTTGCGAACCAAAAAGT	56	559
	R:CATCCAGTTCAACAGTAGTCTTCTC		
*RPL10*	F:CACGGTTCTGTCGTGGTGTA	58	459
	R:AGCCCCATTTCTTGGAGACG		
*GAPDH*	F:TCCAAAATCGGTATCAACGG	57	970
	R:TGAGATCGATGACGCGGT		
*EF1-α*	F:GAGGCACGCTACGAGGAGATTA	59	767
	R:GGGAACTCCTGGAAAGACTCCA		

**Table 2:** Primers of reference genes for RT-qPCR in *S. trifolii*.

Gene	Primer Sequences (5′–3′)	Tm (°C)	Length (bp)	Eff (%)	R^2^	Standard Curve Equation
*TUB*	F:GGCGATGGCGGTGGTGTTAGAC	60	108	90.951	0.999	Y = −3.560x + 42.985
	R:GGATTCAAGGTGGGCATCAACT					
*β-actin*	F:GTATCCTCACGCTCAAGTA	60	89	94.115	0.990	Y = −3.353x + 40.171
	R:GTTGTAGAAGGTGTGGTG					
*RPL9*	F:GAGTGCTGAAGGTAGAGAA	60	161	108.777	0.990	Y = −3.128x + 37.734
	R:AGTGGTGACACAGTTGAT					
*RPL10*	F:GCAGTTCTTGACGAGGTA	60	87	90.799	0.994	Y = −3.564x + 41.675
	R:CTGGTGTCTGACGAGTAC					
*GAPDH*	F:TGCCAAGAAGGTCATCAT	60	79	94.201	0.990	Y = −3.469x + 49.458
	R:GTCGTATGCCTCAAGGTT					
*EF1-α*	F:GCGATCCAGCCCCCTTC	60	119	102.27	0.992	Y = −3.269x + 46.702
	R:TTGAGGATGCCGGTCTCAAC					

Eff, RT-qPCR efficiency; R^2^, regression coefficient; F, forward primer; R, reverse primer.

## Data Availability

The original contributions presented in this study are included in the article/Supplementary Material. Further inquiries can be directed to the corresponding author.

## Supplementary Materials

The following supporting information can be downloaded at: https://www.mdpi.com/article/10.3390/insects16050527/s1. Figure S1: Amplification melting curves of primers for six reference genes of *Scotogramma trifolii* (Lepidoptera: Lepidoptera) in RT-qPCR.
