# Supplementary material for: Selection and Validation of Stable Reference Genes for RT-qPCR in Scotogramma trifolii (Lepidoptera: Noctuidae)
Source: Insects. 2025 May 15;16(5):527. doi: 10.3390/insects16050527 (PMC12112379; doi:10.3390/insects16050527)
Supplement: Supplementary file 1 [file insects-16-00527-s001.zip › insects-3596038-supplementary.pdf]

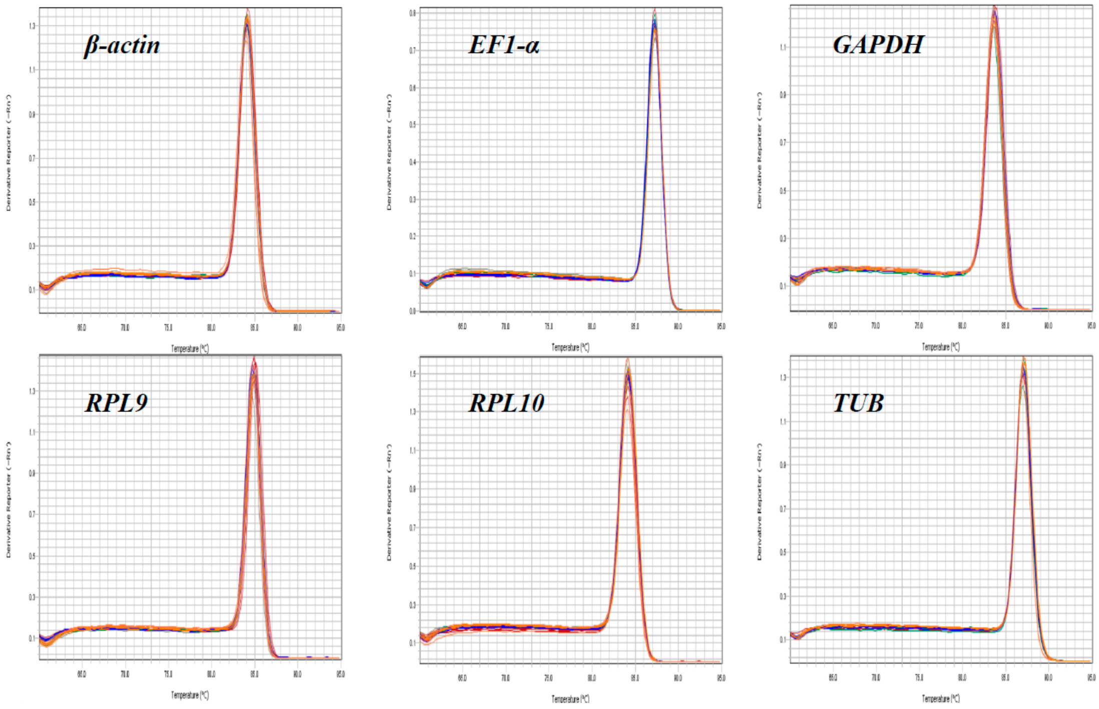


Figure S1 Amplification melting curves of primers for six reference genes of *Scotogramma trifolii* (Lepidoptera: Lepidoptera) in RT-qPCR.
